# Clinical risk factors for increased respiratory drive in intubated hypoxemic patients

**DOI:** 10.1186/s13054-023-04402-z

**Published:** 2023-04-11

**Authors:** Elena Spinelli, Antonio Pesenti, Douglas Slobod, Carla Fornari, Roberto Fumagalli, Giacomo Grasselli, Carlo Alberto Volta, Giuseppe Foti, Paolo Navalesi, Rihard Knafelj, Paolo Pelosi, Jordi Mancebo, Laurent Brochard, Tommaso Mauri

**Affiliations:** 1 Department of Anesthesia, Critical Care and Emergency, Fondazione IRCCS Ca’ Granda Ospedale Maggiore Policlinico, Milan, Italy; 2 Department of Pathophysiology and Transplantation, University of Milan, Milan, Italy; 3 Department of Critical Care Medicine, McGill University, Montreal, QC Canada; 4 Research Centre On Public Health, University of Milano – Bicocca, Monza, Italy; 5 Anesthesia and Critical Care Service 1, Niguarda Hospital, Milan, Italy; 6 Morphology, Surgery and Experimental Medicine, Anesthesia and Intensive Care Unit, University of Ferrara, Ferrara, Italy; 7 Anesthesia and Critical Care, San Gerardo Hospital, ASST Monza, Monza, Italy; 8 Anesthesia and Intensive Care, Department of Medicine – DIMED, Padua University Hospital, University of Padua, Padua, Italy; 9 Center for Internal Intensive Medicine (MICU), University Medical Center Ljubljana, Ljubljana, Slovenia; 10 Anesthesia and Intensive Care, San Martino Policlinico Hospital, IRCCS for Oncology and Neurosciences, Genoa, Italy; 11 Department of Surgical Sciences and Integrated Diagnostics, University of Genoa, Genoa, Italy; 12 Interdepartmental Division of Critical Care Medicine, University of Toronto, Toronto, Canada; 13 Hospital de la Santa Creu i Sant Pau, Barcelona, Spain

**Keywords:** Risk factors, Respiratory drive, Acute respiratory failure, Positive end-expiratory pressure

## Abstract

**Background:**

There is very limited evidence identifying factors that increase respiratory drive in hypoxemic intubated patients. Most physiological determinants of respiratory drive cannot be directly assessed at the bedside (e.g., neural inputs from chemo- or mechano-receptors), but clinical risk factors commonly measured in intubated patients could be correlated with increased drive. We aimed to identify clinical risk factors independently associated with increased respiratory drive in intubated hypoxemic patients.

**Methods:**

We analyzed the physiological dataset from a multicenter trial on intubated hypoxemic patients on pressure support (PS). Patients with simultaneous assessment of the inspiratory drop in airway pressure at 0.1-s during an occlusion (*P*_0.1_) and risk factors for increased respiratory drive on day 1 were included. We evaluated the independent correlation of the following clinical risk factors for increased drive with *P*_0.1_: severity of lung injury (unilateral vs. bilateral pulmonary infiltrates, PaO_2_/FiO_2_, ventilatory ratio); arterial blood gases (PaO_2_, PaCO_2_ and pHa); sedation (RASS score and drug type); SOFA score; arterial lactate; ventilation settings (PEEP, level of PS, addition of sigh breaths).

**Results:**

Two-hundred seventeen patients were included. Clinical risk factors independently correlated with higher *P*_0.1_ were bilateral infiltrates (increase ratio [IR] 1.233, 95%CI 1.047–1.451, *p* = 0.012); lower PaO_2_/FiO_2_ (IR 0.998, 95%CI 0.997–0.999, *p* = 0.004); higher ventilatory ratio (IR 1.538, 95%CI 1.267–1.867, *p* < 0.001); lower pHa (IR 0.104, 95%CI 0.024–0.464, *p* = 0.003). Higher PEEP was correlated with lower *P*_0.1_ (IR 0.951, 95%CI 0.921–0.982, *p* = 0.002), while sedation depth and drugs were not associated with *P*_0.1_.

**Conclusions:**

Independent clinical risk factors for higher respiratory drive in intubated hypoxemic patients include the extent of lung edema and of ventilation-perfusion mismatch, lower pHa, and lower PEEP, while sedation strategy does not affect drive. These data underline the multifactorial nature of increased respiratory drive.

**Supplementary Information:**

The online version contains supplementary material available at 10.1186/s13054-023-04402-z.

## Background

Respiratory drive is the source signal for a descending cascade which ultimately generates the inspiratory effort and breathing pattern [1]. The neural output from the brainstem centers determines the frequency, velocity, and magnitude of respiratory muscle contraction and thus the rate, flow, and magnitude of tidal ventilation [2].

Biochemical inputs from central [3] and peripheral [4] chemoreceptors (sensing alterations of pCO_2_, pH and oxygen) and various inputs from lung mechanoreceptors and chemoreceptors (affected by changes in lung mechanics, edema and inflammation) [5], together with “behavioral” factors (agitation, anxiety) modulate the activity of the respiratory centers. All these alterations represent hallmark physiological derangements of patients with acute hypoxemic respiratory failure (AHRF), potentially increasing activation of respiratory drive [6]. However, both respiratory drive and most of its physiological determinants (apart from arterial gas analysis) cannot be directly measured at the bedside.

The airway occlusion pressure at 100 ms (*P*_0.1_) [7] is an accurate surrogate for respiratory drive output in mechanically ventilated patients [8]. Moreover, clinical risk factors correlated with determinants of respiratory drive such as the severity of lung injury (e.g., extent of radiologic pulmonary infiltrates), inflammation, the development of organ dysfunction (e.g., the SOFA score) and the brain cortical activity (e.g., RASS score) can be assessed at the bedside. A few small clinical studies have explored the association between clinical risk factors and respiratory drive, and a lack of correlation between deep sedation and lower *P*_0.1_ has recently been described [9].

In terms of clinical interventions, adjustment of ventilation settings is one of the most implemented strategies to modulate respiratory drive and achieve physiological targets [10, 11]. Previous small physiological studies showed that the level of pressure support and addition of sigh breaths affect the *P*_0.1_, tidal volume and respiratory rate of intubated patients [12]. It has also been suggested that higher positive end-expiratory pressure (PEEP) might reduce effort and improve lung protection in animal models during assisted ventilation [13–15], but its effect on respiratory drive and effort seems more variable [11, 16]. Thus, lower PS and PEEP, and a lack of sigh breaths could be considered clinical risk factors for increased drive.

We recently conducted a large pilot randomized controlled trial on the feasibility and safety of addition of intermittent sigh breaths to pressure support in intubated hypoxemic patients [17]. In the present study, we analyzed potential independent correlations between clinical risk factors for increased respiratory drive and *P*_0.1_ on the first day of enrolment.

## Methods

### Patients, study design and setting

We analyzed the dataset obtained from an international, multicentered, randomized controlled trial (NCT03201263) [17] in order to explore respiratory physiology, as pre-planned in the original study protocol [18]. The trial included mechanically ventilated patients with acute hypoxemic respiratory failure (PaO_2_/FiO_2_ ≤ 300) who had been intubated for 7 days or less and who had been switched to pressure support ventilation within the prior 24 h. After enrollment and randomization, physiological measurements including *P*_0.1_ were collected daily. From the original database, in the present study we included all patients with a measurement of *P*_0.1_ on day 1.

The study was approved by the Ethics Committee of Fondazione IRCCS Ca’ Granda Ospedale Maggiore Policlinico of Milan, Italy (ref. 318/2017). The institutional review boards of all participating centers approved the study. Informed consent was obtained for each patient following local regulations. Additional details regarding exclusion criteria and methods of the original study have been described previously [18].

### Variables and measurements

We analyzed measurements of *P*_0.1_ and clinical risk factors for increased respiratory drive collected in each patient at the same time on day 1. Measurements of *P*_0.1_ were performed by the built-in software of each ventilator (see Additional file 1 for brands and models) and recorded in the online form by clinicians. We collected different variables for each category of clinical risk factors, as outlined by our previous work [6] and further defined below, in the statistical analysis section: severity of lung injury, arterial blood gases, sedation, and systemic activation of inflammation. The following mechanical ventilation settings were also collected: PS level, external set PEEP and addition of sigh. Note that PS level and set PEEP has been titrated as follow, according to the original study protocol: at least every 8 h, the PSV level was adjusted to maintain a tidal volume of 6–8 mL/kg PBW and respiratory rate of 20–35 bpm, while PEEP and FiO_2_ were managed to keep the SpO_2_ at 90–96%.

Demographic characteristics (age, sex, body mass index), etiology of AHRF and clinical severity at enrollment were also included in the analysis. Additional details about data collection can be found in Additional file 1.

### Statistical analyses

Normally distributed data are described with mean and standard deviation (SD), whereas non-normally distributed data are described using median and quartiles [*Q*_1_–*Q*_3_]. Descriptive statistics are used to characterize the study population. A two-tailed *p*-value below 0.05 was considered as statistically significant.

The bivariate relationship between *P*_0.1_ values and clinical risk factors was assessed using a generalized linear model based on a gamma distribution with a log link function, because *P*_0.1_ was a continuous variable with no zero values and a right skewed distribution. The following candidate clinical risk factors were assessed in the bivariate analyses: severity of lung injury (diagnosis of ARDS [categorical], PaO_2_/FiO_2_ and ventilatory ratio); arterial blood gases (PaO_2_, PaCO_2_ and pHa); sedation depth measured by the Richmond Agitation-Sedation Scale (RASS) [19] [categorical], and sedative drugs defined according to the type and number of different classes of drugs administered for sedation (sedative/anesthetics, opioids, both sedative and opioids, none; see Additional file 1) [categorical]; activation of systemic inflammation (SOFA score [categorical] and lactate); ventilation settings (pressure support, set PEEP and addition of sigh breaths). Then, we constructed a multivariate model with a stepwise approach to identify independent clinical risk factors for *P*_0.1_.

Physiologically sound clinical risk factors were included in the stepwise multivariate approach, while baseline characteristics (age, sex, and SAPS II at admission) and PaCO_2_ were considered adjusting factors, so as fixed effects of the multivariate model. PaCO_2_ was considered only as an adjusting factor and not as a clinical risk factor due to its inverse bivariate association with *P*_0.1_, suggesting that it represents a consequence of increased drive. Multicollinearity was tested to exclude high intercorrelation among the determinants included in the final multivariate regression model.

Results of bivariate regression models were reported as *β* coefficient and *p*-value, while for the multivariate model we also reported increase ratio (IR) estimates as exp(*β*) with 95% confidence intervals (95%CI). IR is the relative increase of *P*_0.1_ at one unit increase of the clinical risk factor.

Statistical analyses were performed with SAS 9.4 TS Levek 1M7 (2020 SAS Institute Inc., Cary, NC, USA) and R Studio 2002.07.1 (2009-2002Rstudio PBC).

## Results

Two-hundred-seventeen patients with measurements of *P*_0.1_ and its potential clinical risk factors simultaneously recorded on study day 1 were included in the analysis. The median *P*_0.1_ was 1.5 cmH_2_O with a range of 0.1–8.5 cmH_2_O. These values are in line with those described in previous smaller series [7–9] and indicate a wide range of respiratory drive activation.

The main characteristics of the study population are reported in Table 1: 71% patients were male, and median time from intubation was 3 days. The admitting diagnosis was infectious pneumonia in 58% of patients and 47% fulfilled diagnostic criteria for ARDS, with the remaining having AHRF with unilateral infiltrates on chest x-ray.

**Table 1:** Baseline demographics and clinical characteristics

	All patients (*n* = 217)
Demographics	
Men, No. (%)	153 (71)
Age, years	65 [53–75]
BMI, kg/m^2^	26 [23–29]
Recent medical history	
Intubation days, median [Q1–Q3]	3 [2–5]
SAPS II, median [Q1–Q3]	41 [31–52]
Etiology	
Pneumonia, No. (%)	127 (58)
Aspiration of gastric content, No. (%)	20 (9)
Non-pulmonary sepsis, No. (%)	37 (17)
Other^, No. (%)	54 (25)
Lung injury	
Bilateral Infiltrates (ARDS diagnosis) No. (%)	102 (47)
PaO_2_/FiO_2_, mmHg	228 [190–254]
Clinical status and ventilation settings on day 1	
SOFA	6 [4–8]
RASS	− 1 [− 1 to 0]
PEEP, cmH_2_O	8 [7–10]
Pressure support, cmH_2_O	8 [6–12]
Addition of sigh breaths, No. (%)	109 (50)
FiO_2_	0.4 [0.3–0.4]

Data are expressed as median [*Q*_1_–*Q*_3_] or as number (%), as appropriate

BMI: Body mass index; SAPS II: Simplified Acute Physiology Score II; ARDS: acute respiratory distress syndrome; SOFA: Sequential Organ Failure Assessment Score; RASS: Richmond Agitation Sedation Scale; PEEP: positive end-expiratory pressure

**^“**Other” includes lung contusion, lung vasculitis, drowning, pancreatitis, severe burns, major trauma, TRALI or other conditions

### Association between potential clinical risk factors for increased respiratory drive and *P*_0.1_

As expected, several candidate factors were correlated with *P*_0.1_ at bivariate analysis, indicating the overlapping interconnections between clinical risk factors and physiological determinants of respiratory drive. We report here the main findings, while additional figures can be found in Additional file 1.

#### Severity of lung injury

**Fig. 1:**
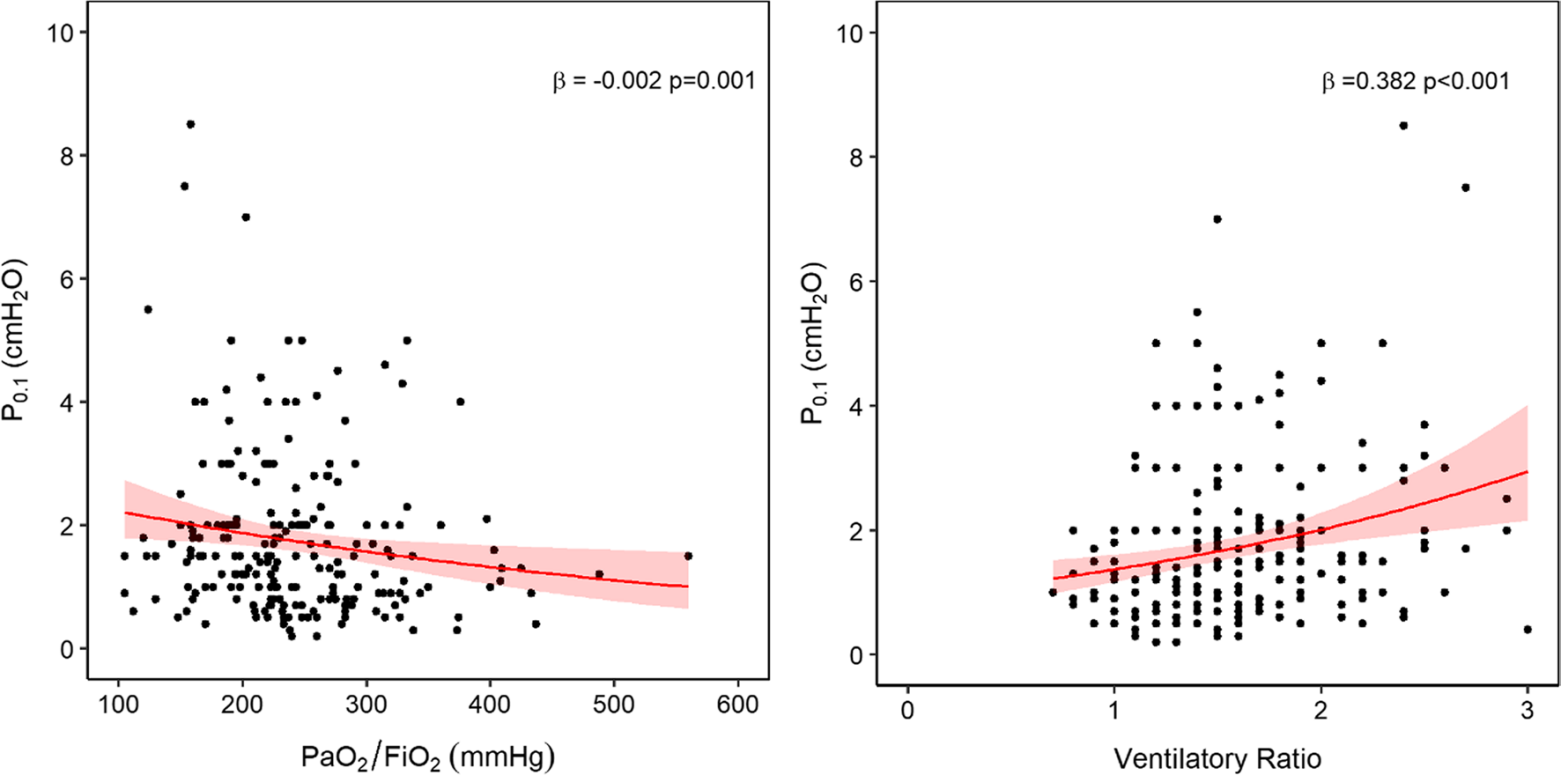
Association between severity of lung injury (ventilation/perfusion mismatch) and *P*_0.1_. Results of bivariate analyses show that *P*_0.1_ was inversely associated to PaO_2_/FiO_2_ and directly associated with ventilatory ratio, indicating that impairment in oxygenation and CO_2_ clearance are clinical risk factors for increased respiratory drive

Diagnosis of ARDS versus presence of unilateral infiltrates was associated with higher *P*_0.1_ (*β* = 0.22, *p* = 0.014) (Additional file 1: Fig. E1). PaO_2_/FiO_2_ was inversely associated with *P*_0.1_ (*β* =  − 0.002, *p* = 0.001) while the ventilatory ratio was directly associated (*β* = 0.382, *p* < 0.001) (Fig. 1). To further investigate the role of ventilatory ratio, we explored the correlation between *P*_0.1_ and minute ventilation (*β* = 0.096, *p* < 0.001), respiratory rate (*β* = 0.047, *p* < 0.001) and tidal volume (*β* =  − 0.0002, *p* = 0.582) (Additional file 1: Fig. E2).

**Fig. 2:**
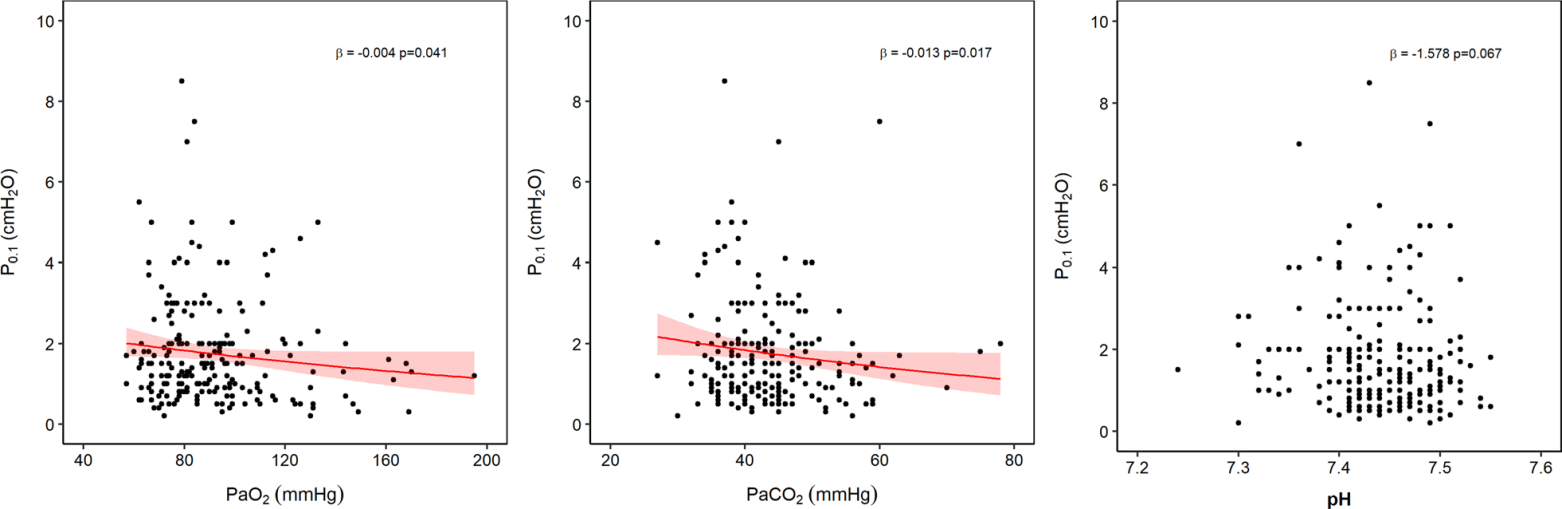
Association between arterial blood gases and *P*_0.1_. Results of bivariate analyses show that *P*_0.1_ was inversely associated with both PaO_2_ and PaCO_2_, while the association with arterial pH was not statistically significant

#### Arterial blood gases

There was a significant association between lower PaO_2_ (*β* =  − 0.004, *p* = 0.041) and, counterintuitively, lower PaCO_2_ (*β* =  − 0 to 013, *p* = 0.017) with higher *P*_0.1_. The correlation between pHa and *P*_0.1_ did not reach statistical significance (*β* =  − 1.578, *p* = 0.067) (Fig. 2).

#### Sedation

**Fig. 3:**
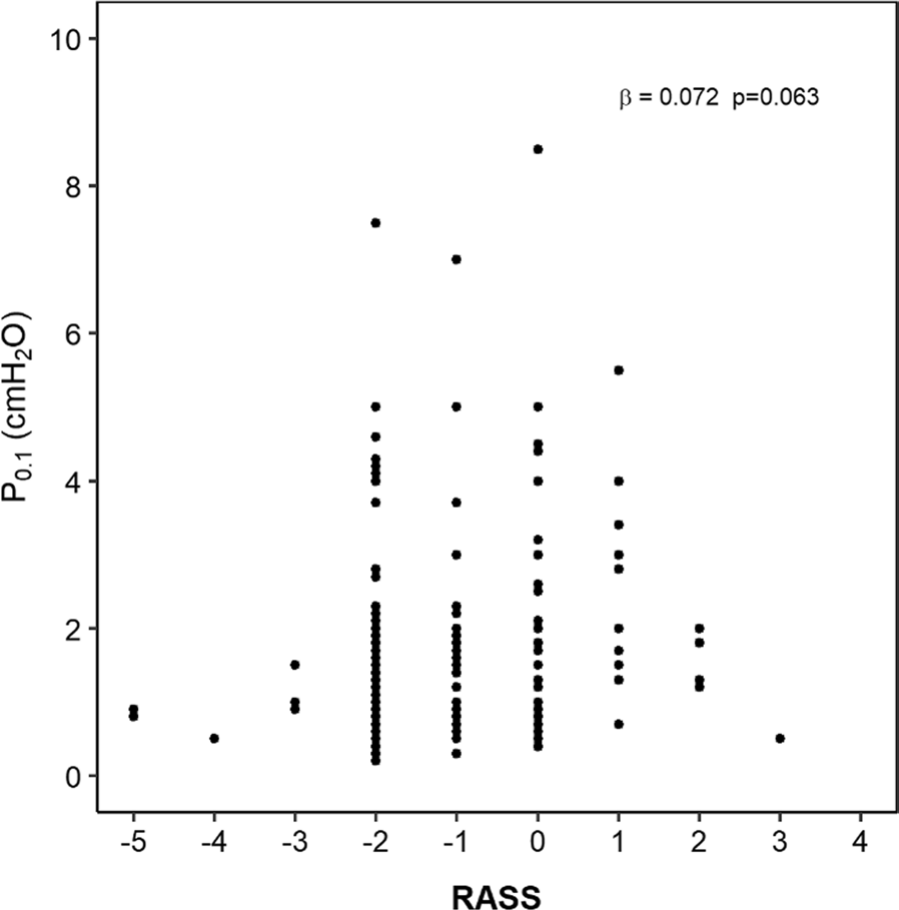
Impact of sedation depth on *P*_0.1_. No significant association was found between *P*_0.1_ and RASS category at bivariate analysis

The correlation between higher RASS category and higher *P*_0.1_ did not reach statistical significance (*β* = 0.072, *p* = 0.063) (Fig. 3); moreover, the type of drug used for sedation did not appear to influence *P*_0.1_ (Additional file 1: Fig. E3).

#### Activation of systemic inflammation

Neither SOFA score (*β* = 0.006, *p* = 0.664) nor arterial lactates (*β* = 0.005, *p* = 0.846) were associated with *P*_0.1_ (Additional file 1: Fig. E4).

**Fig. 4:**
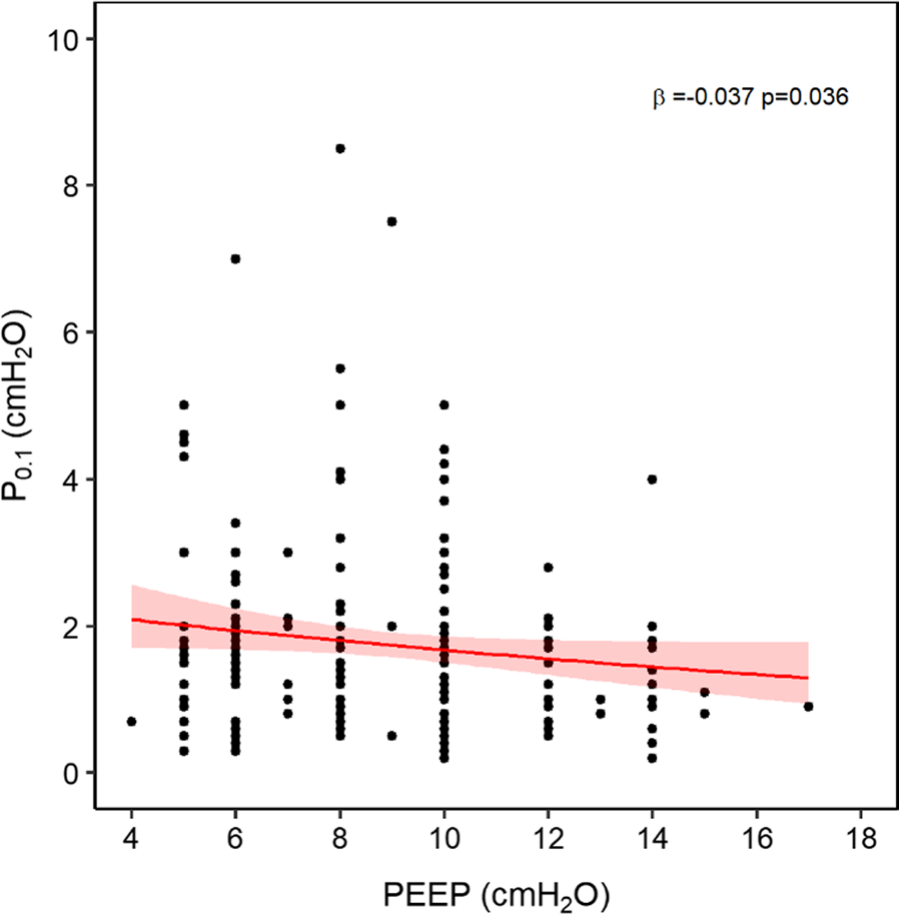
Impact of PEEP on *P*_0.1_. Clinically set PEEP was inversely associated with *P*_0.1_, indicating that lower PEEP is a risk factor for increased respiratory drive in these patients

#### Ventilation settings

Clinically set PEEP was inversely correlated with *P*_0.1_ (*β* =  − 0.04, *p* = 0.036) (Fig. 4), while the level of pressure support was not associated with respiratory drive (*β* =  − 0.008, *p* = 0.526). Application of sigh breaths had no impact on *P*_0.1_ (*β* = 0.055, *p* = 0.542) (Additional file 1: Fig. E5).

### Independent clinical risk factors correlated with increased respiratory drive

Results from the multivariate analysis investigating the independent impact of clinical risk factors on higher *P*_0.1_ are presented in Table 2. Based on previous physiological data and reasoning, the following factors were included as predictors in the model, adjusted for age, sex, SAPS II score and PaCO_2_: diagnosis of ARDS vs. unilateral lung injury, PaO_2_/FiO_2_ ratio, ventilatory ratio, PaO_2_, pHa, RASS, SOFA, sedative drugs, PS level, PEEP and addition of sigh breaths.

**Table 2:** Multivariate regression model describing independent clinical risk factors for increased *P*_0.1_

Variable	*Β*	Increase ratio*	95% Wald confidence interval	*P* value
Determinants of respiratory drive				
PaO_2_/FiO_2_ (mmHg)	− 0.002	0.998	0.997–0.999	0.004
Ventilatory ratio	0.431	1.538	1.267–1.867	< 0.001
Bilateral (ARDS) versus unilateral infiltrates	0.209	1.233	1.047–1.451	0.012
pHa	− 2.260	0.104	0.024–0.464	0.003
Clinically set PEEP (cmH_2_O)	− 0.050	0.951	0.921–0.982	0.002
Adjusting factors				
Female versus male sex	− 0.244	0.784	0.658–0.933	0.006
Age (years)	− 0.004	0.996	0.990–1.001	0.123
SAPS II score	− 0.004	0.996	0.991–1.001	0.138
PaCO_2_ (mmHg)	− 0.027	0.973	0.963–0.984	< 0.001

ARDS: acute respiratory distress syndrome; PEEP: positive end-expiratory pressure; PS: pressure support; SAPS II: Simplified Acute Physiology Score II

*Increase ratio = exp(*β*)

We identified the following the following clinical parameters independently associated with increased risk of higher *P*_0.1_: diagnosis of ARDS (increase ratio: 1.233 [95%CI 1.047–1.451]), lower PaO_2_/FiO_2_ ratio (IR 0.998 [0.997–0.999], higher ventilatory ratio (1.538 [1.267–1.867], lower pH (IR 0.104 [0.024–0.464]) and lower set PEEP (0.951 [0.921–0.982]) (Table 2).

RASS, SOFA, sedative drugs, PS level and addition of Sigh were not significantly associated with *P*_0.1_.

## Discussion

This study investigated clinical risk factors for increased *P*_0.1_ in a large population of intubated patients with AHRF undergoing pressure support ventilation. Independent factors predicting higher respiratory drive measured by *P*_0.1_ were diagnosis of ARDS, lower PaO_2_/FiO_2_, higher ventilatory ratio, lower arterial pH, and lower clinically set PEEP. Sedation strategy (target RASS and drugs type), instead, was not associated with modulation of respiratory drive.

In patients intubated for AHRF, high respiratory drive may hinder safe spontaneous breathing during assisted ventilation by inducing high lung stress and occult pendelluft [20–22], dyssynchronies [23] and dyspnea [24]. Stimuli related to the severity of lung injury, including impairment of gas exchange and altered respiratory mechanics, but also activation of peripheral lung receptors by edema or inflammation, may lead to increased drive [6]. In addition, extra-pulmonary factors such as agitation, systemic inflammation and metabolic acidosis may contribute. In clinical practice, most of the determinants stimulating the respiratory centers are impossible to measure. However, several clinical risk factors measured at the bedside could reflect these inputs and thus be associated with *P*_0.1_. Understanding the impact and the independent contribution of these factors in determining the value of *P*_0.1_ could be useful to guide safe initiation and management of assisted ventilation in patients with AHRF [10]. On the other hand, lack of association between a candidate clinical risk factor and *P*_0.1_ could be interpreted in two ways: either the clinical risk factor is not an accurate surrogate for the physiological determinant, or the clinical factor has limited relevance in patients.

The present study describes that in patients with AHRF, the severity of lung injury, as assessed by larger extent of pulmonary infiltrates, lower oxygenation, and higher ventilatory ratio, is a clinical risk factor for increased *P*_0.1_. More specifically, the extent of lung edema and of ventilation/perfusion mismatch could represent global markers of structural and functional impairment of the lung [25], associated with higher *P*_0.1_.

In healthy subjects, respiratory drive mainly depends on the chemoreflex control of arterial CO_2_. On the contrary, assisted ventilation limits the spontaneous modulation of tidal volume in response to the chemical feedback from CO_2_ [26, 27]. Indeed, PaCO_2_ seems to be a consequence more than a determinant of drive and effort in our patients [20, 28], given the correlation between higher *P*_0.1_ and both higher minute ventilation and lower PaCO_2_. In this perspective, since the minute ventilation enters in the calculation of ventilatory ratio, the positive association between ventilatory ratio and *P*_0.1_ could have been driven by the effect of higher minute ventilation.

The correlation between PaO_2_ and P_0.1_ at bivariate analysis likely reflects patient severity, but PaO_2_ was not confirmed as an independent risk factor for elevated *P*_0.1_. This finding might also depend on the protocol adopted for titration of SpO_2_ in this study, resulting in a limited range of values for PaO_2_. Although it has been demonstrated that moderate decreases in PaO_2_ could increase respiratory drive in some patients [29], it is known that the effect of PaO_2_ becomes much stronger below 60 mmHg [30].

Similar to a recent study [9], we could not find an independent correlation between sedation depth and sedative drug type on *P*_0.1_, suggesting a limited effect of sedatives and opioids in the modulation of respiratory drive in patients with AHRF, as compared to pulmonary and systemic disease severity. However, the lack of correlation between sedation depth and drive might also be due to the limited ability of the RASS score to evaluate descending cortical input to the respiratory centers.

Arterial lactate was not a clinical risk factor for increased drive in this study, in contrast with our previous finding in septic patients without acute respiratory failure [31]. The lack of correlation between *P*_0.1_ and both lactate and SOFA score could suggest that the role of extra-pulmonary organ failure and distal hypoperfusion may have less of an impact on respiratory drive when lung injury is present.

Adjustment of ventilation settings is probably the most common clinical intervention used to modulate respiratory drive and effort when attempting to achieve lung and diaphragm protective ventilation [10]. Early studies showed that changing the level of support and PEEP can influence the breathing pattern [32]. Indeed, increasing the level of inspiratory assist decreases respiratory drive and effort [33] by unloading the respiratory muscles in patients recovering from AHRF [34–37]. However, it is now recognized that a significant number of patients with AHRF may not exhibit such a response [38], suggesting the presence of high respiratory drive due to stimuli other than arterial pH and PaCO_2_ [21, 39]. Indeed, we could not find a correlation between the level of support and *P*_0.1_. Interestingly, our results show that higher PEEP is associated with lower *P*_0.1_. This finding reinforces the accumulating experimental [15] and clinical [40] evidence of the beneficial effects of higher PEEP during spontaneous breathing, likely due to the modulation of respiratory drive and effort induced by stabilizing alveolar recruitment [11, 16].

The strengths of the present analysis are the large multicenter sample of patients with AHRF, the early timing of assessment after switching to assisted ventilation and the accurate collection of physiological and clinical data from a randomized controlled trial. Our study also has limitations. First, automated *P*_0.1_ measurements overall underestimate absolute *P*_0.1_ values with differences between different ventilators [8, 41]. This can also be seen as a strength as our data coincide with those available in clinical practice, which rely on *P*_0.1_ displayed by different ventilators. Second, we lack measurements of respiratory system compliance or recruitment and thus we can only hypothesize about the mechanisms by which PEEP modulates drive. Third, we could only analyze some of the potential clinical risk factors for increased respiratory drive, while other factors like pulmonary and systemic inflammatory cytokines were not collected.

## Conclusions

In a large population of intubated hypoxemic patients, clinical risk factors independently associated with higher *P*_0.1_ included the extent of pulmonary infiltrates, the degree of ventilation/perfusion mismatch and lower arterial pH. Higher set PEEP was independently associated with lower *P*_0.1_. Sedation strategy, including actual RASS score and sedative drug type, despite being extensively used in clinical practice to control drive, seems to have no impact on *P*_0.1_. These results confirm the multifactorial nature of the activation of respiratory drive and highlight the key role of severity of lung injury in increasing drive.

### Supplementary Information

**Additional file 1.** Clinical risk factors for increased respiratory drive in intubated hypoxemic patients: additional methods and results.

## Data Availability

The datasets used and/or analyzed during the current study are available from the corresponding author on reasonable request.

## Abbreviations

AHRF: Acute hypoxemic respiratory failure
ARDS: Acute respiratory distress syndrome
CI: Confidence interval
IR: Increase ratio
P0.1: Inspiratory drop in airway pressure at 0.1-s during an occlusion
PaCO_2_: Partial pressure of arterial CO_2_
PaO_2_/FiO_2_: Partial pressure of arterial O_2_/fraction of inspired O_2_
PEEP: Positive end expiratory pressure
PS: Pressure support
RASS: Richmond Agitation Sedation Scale
SAPS: Simplified Acute Physiology Score
SD: Standard deviation
SOFA: Sequential Organ Failure Assessment score
