# Supplementary material for: Clinical risk factors for increased respiratory drive in intubated hypoxemic patients
Source: Crit Care. 2023 Apr 11;27:138. doi: 10.1186/s13054-023-04402-z (PMC10088111; doi:10.1186/s13054-023-04402-z)

**Clinical risk factors for increased respiratory drive in intubated hypoxemic patients**

Elena Spinelli, Antonio Pesenti, Douglas Slobod, Carla Fornari, Roberto Fumagalli, Giacomo Grasselli, Carlo Alberto Volta, Giuseppe Foti, Paolo Navalesi, Rihard Knafelj, Paolo Pelosi, Jordi Mancebo, Laurent Brochard, Tommaso Mauri

**Online Supplement**

**Additional Methods**

**Study design and population of the PROTECTION Trial**

The PROTECTION Trial was conducted between December 2017 and May 2019 at the ICUs of 20 hospitals from eight countries. The trial enrolled patients with AHRF (PaO2/FIO2 ratio ≤ 300 mmHg and clinical PEEP ≥ 5 cm H2O) who had been receiving invasive ventilation for > 24 h and ≤ 7 days and switched to PSV for ≥ 4 and ≤ 24 hours. Patients had to be alert and calm or lightly sedated (Richmond Agitation-Sedation Scale value at enrollment between –2 and 0). The study assessed the feasibility and safety of adding a sigh breath (i.e. a pressure control breath set at total end-inspiratory pressure of 30 cm H2O for a 3-s insufflation time, at a rate of 1-per-minute) to PSV.

**Ventilation management according to the study protocol**

Patients were randomized to receive PSV with addition of sigh (sigh group) or without sigh (no-sigh group). The study protocol prescribed a strategy for titration of PSV settings (PS level and PEEP), which was identical for both the study arms: at least every 8 h, the PSV level was adjusted to maintain a tidal volume of 6 to 8 mL/kg PBW and respiratory rate of 20 to 35 bpm, while PEEP and FIO2 were managed to keep the SpO2 at 90% to 96%. Criteria for failure of PSV and restoration of controlled ventilation were also protocolized.

**Data collection**

The dataset used for the current analysis has been extracted from the database of the PROTECTION trial, which had been derived from the electronic case report forms (eCRF) of the enrolled patients. Daily data reported in the eCRF included vital signs, blood gases, ventilation settings and pattern, use of sedative drugs. They were collected every day between 6:00 and 10:00 in the morning.

**Measurement of P_0.1_**

P_0.1_ was measured using automated maneuvers on ICU ventilators and reported in the eCRF.

P0.1 measurement was performed every day, together with the daily data collection. P_0.1_ values were reported as positive by convention.

The technique to measure P_0.1_ varies between ventilators (1, 2). Since the ventilator specific information were not reported eCRF, it was not possible to know the brand and the model of ventilator used in each patient. However, we retrospectively collected data on the ventilators used at each participating center at the time of patients’ enrollment (see below).

**Sedation depth and drugs**

Sedation depth was reported as the value of Richmond Agitation and Sedation Scale at the time of daily data collection.

Data queries included a pre-specified list of drugs commonly used for sedation in ICU. For the purpose of our analysis, we classified the drugs in two categories:

-sedatives/anesthetics: intravenous propofol, midazolam, ketamine, dexdemetomidine plus oral benzodiazepines

-opioids: morphine, fentanyl, sufentanyl, remifentanyl.

Four classes were then defined based on the type and number of different classes of drugs that they were receiving at day1: only sedatives, only opioids, both or none.

**List of ventilators used in patients enrolled in the PROTECTION Trial:**

Getinge group: Servo-i® and Servo-u®, Solna, Sweden

Dräger: Evita 4®, Evita XL, Infinity V500, Infinity V800, Lübeck, Germany

Covidien: PB 840® and PB850®, Carlsbad, USA

Hamilton Medical: G5®, Bonaduz, Switzerland

Vyaire Medical: Avea Viasys® and Lux iX5®, Illinois, USA

**References**

1. Telias I, Junhasavasdikul D, Rittayamai N, Piquilloud L, Chen L, Ferguson ND, Goligher EC, Brochard L. Airway Occlusion Pressure As an Estimate of Respiratory Drive and Inspiratory Effort during Assisted Ventilation. *Am J Respir Crit Care Med* 2020; 201: 1086-1098.

2. Beloncle F, Piquilloud L, Olivier PY, Vuillermoz A, Yvin E, Mercat A, Richard JC. Accuracy of P0.1 measurements performed by ICU ventilators: a bench study. *Ann Intensive Care* 2019; 9: 104.

**Figure E1. Association between diagnosis of ARDS and high P_0.1_**

Results of bivariate analysis show that diagnosis of ARDS was associated with higher P_0.1_ compared to unilateral infiltrates.

**_
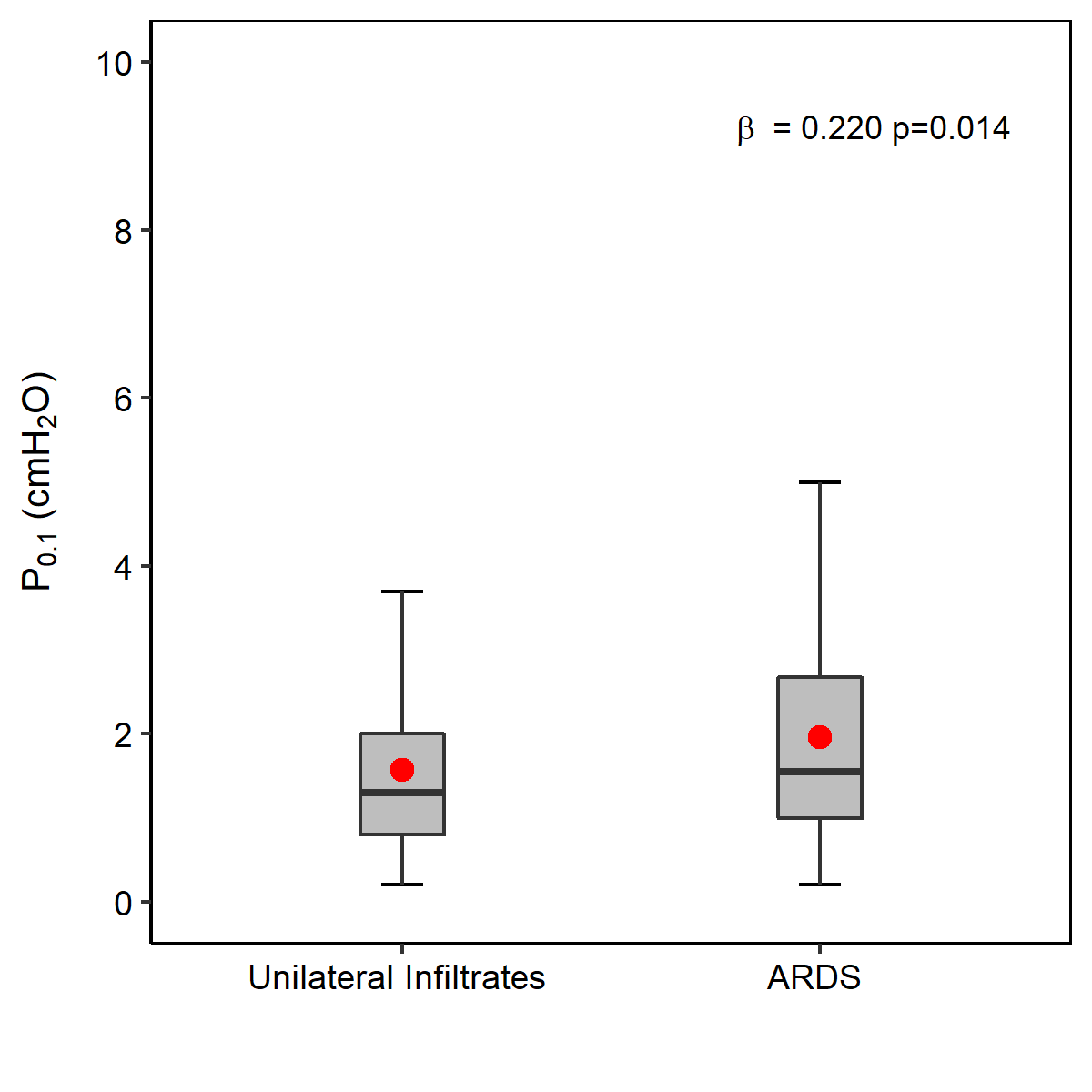
_**

**Figure E5. P_0.1_ and respiratory pattern**

Higher values of P0.1 were associated with higher minute ventilation and higher respiratory rate, but not with tidal volume


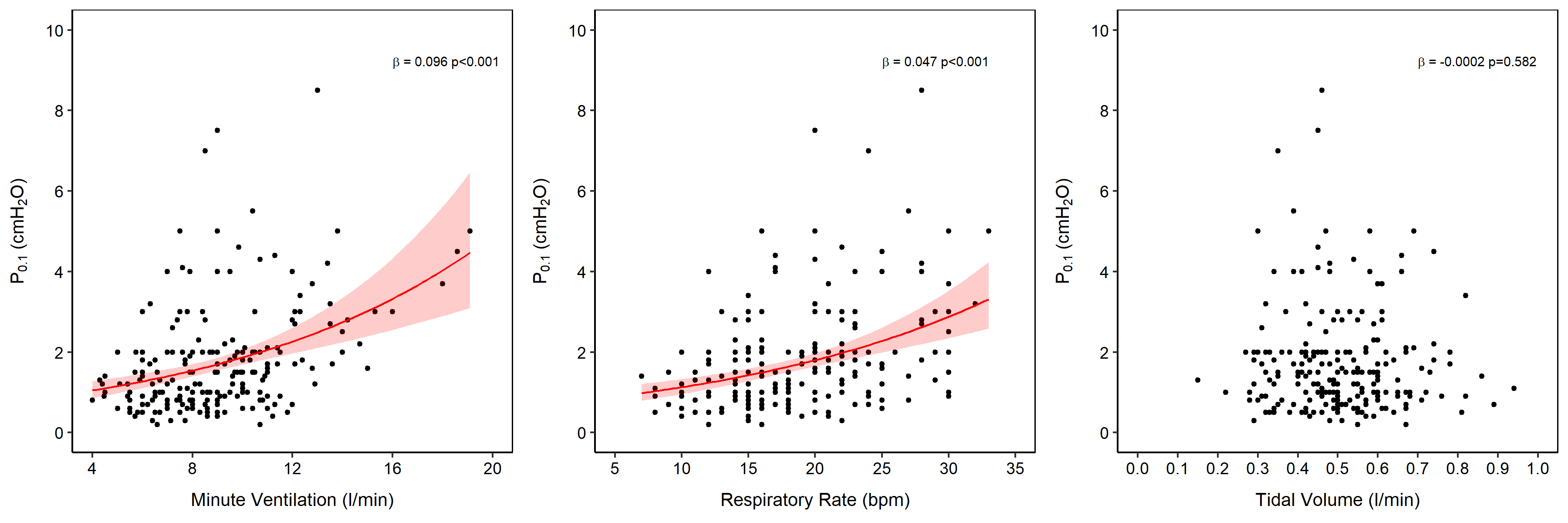


**Figure E3. Lack of association between classes of sedation drugs and P_0.1_**

When patients were categorized according to the class of sedatives (type and number of drugs), no difference were found in terms of P0.1 at bivariate analyses.

**
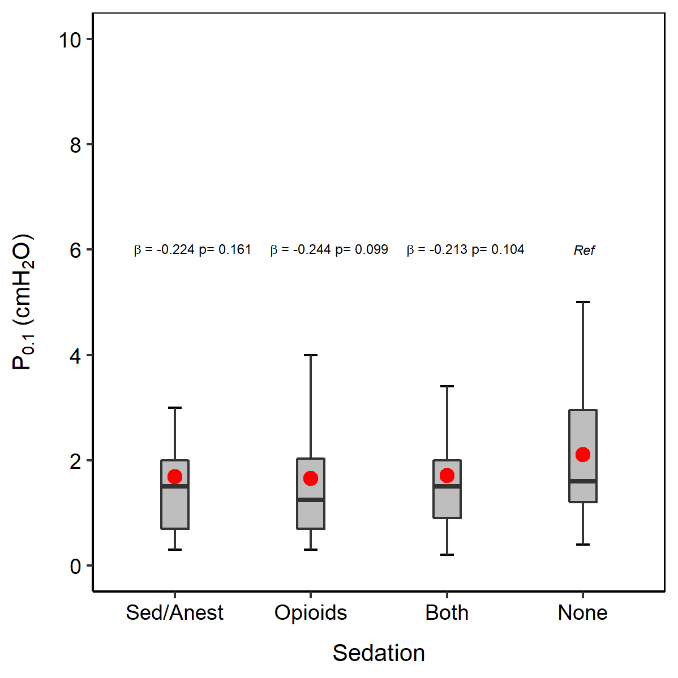
**

**Figure E4. Lack of association between SOFA score and arterial lactates and P_0.1_**

Results of bivariate analysis showed no association between P_0.1_ and SOFA score and P_0.1_ and arterial lactates.

**_
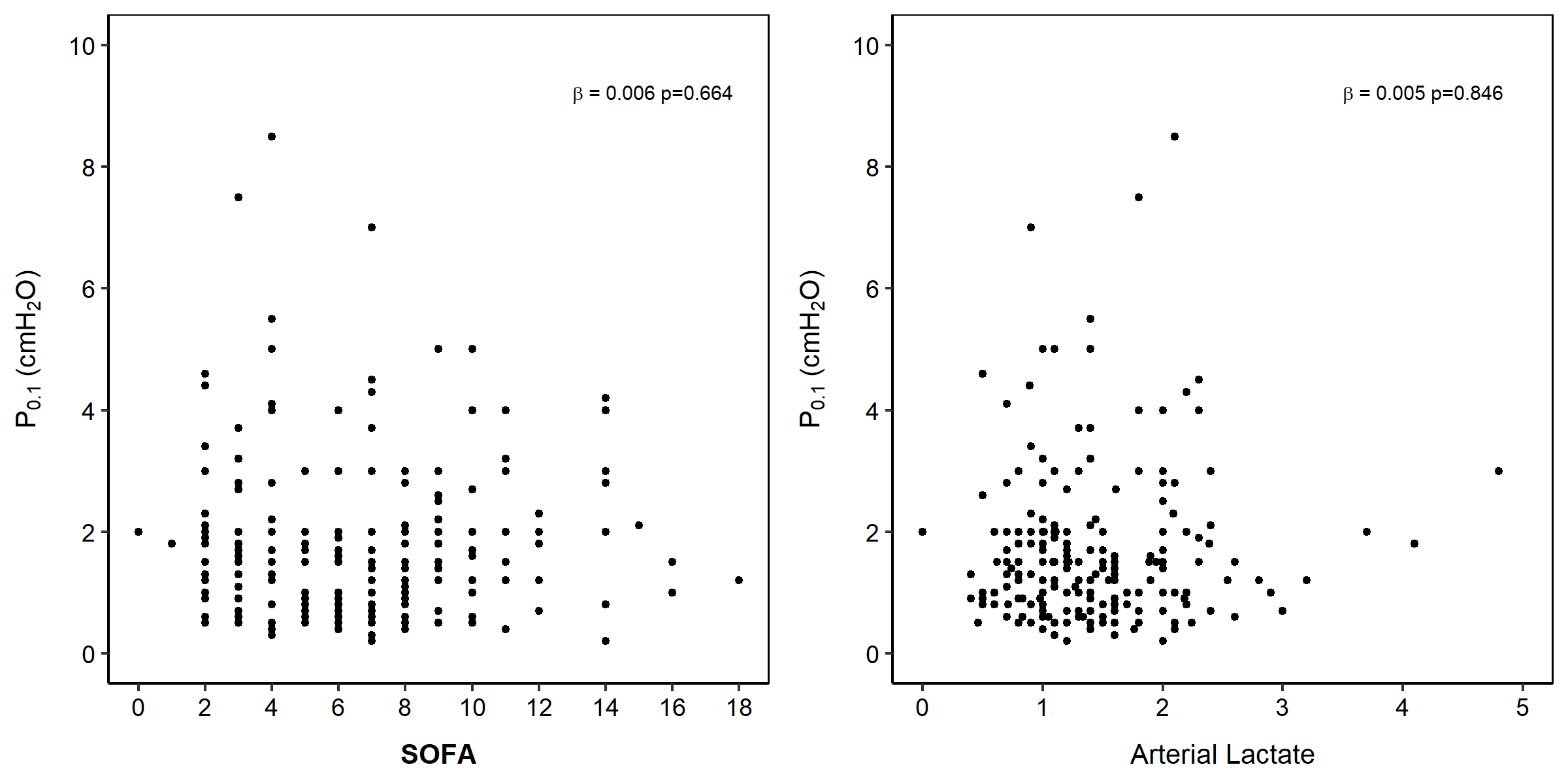
_**

**Figure E5. No impact of PS level and addition of sigh breaths on P_0.1_**

Results of bivariate analysis show no association between level of Pressure Support [continuous] and P_0.1_. Addition of Sigh had no impact on P_0.1._


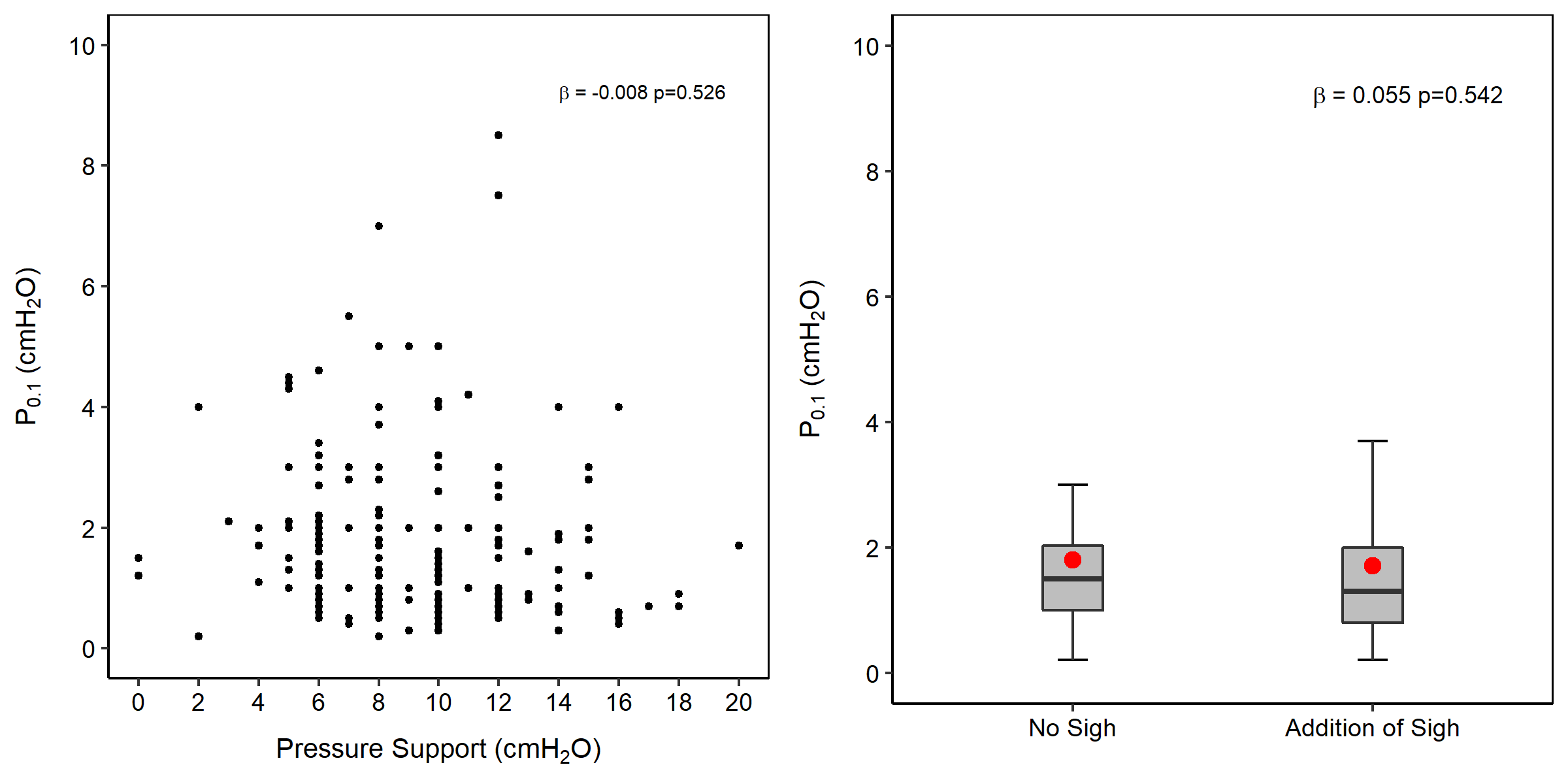

Supplement: Supplementary file 1 — Additional file 1. Clinical risk factors for increased respiratory drive in intubated hypoxemic patients: additional methods and results. [file 13054_2023_4402_MOESM1_ESM.docx]
